# Effects of Different Agents of the Lubrication of i-gel Airway on the Incidence of Postoperative Sore Throat: A Prospective Randomised Controlled Trial

**DOI:** 10.7759/cureus.65278

**Published:** 2024-07-24

**Authors:** Pavithra Balachandran, Ramamurthy Balaji, Dheepak Kumaran, Balasubramaniam Gayathri

**Affiliations:** 1 Anaesthesiology, SRM Medical College Hospital and Research Centre, Chennai, IND

**Keywords:** postoperative sore throat, lmas, general anesthesia, i-gel, lubricants

## Abstract

Background and aim: The aim of this study is to compare the effect of different lubricating agents used with the i-gel® laryngeal mask airway on the incidence of postoperative sore throat.

Materials and methods: After obtaining institutional ethics approval, this prospective trial was conducted on 150 patients who were scheduled for minor surgical procedures. The study population was placed in three groups of 50 each, after randomization with computer-generated random numbers, based on the lubricant used (Group LJ: with lignocaine jelly as the lubricant, Group WJ: with water-based jelly as a lubricant, Group NS: with 0.9% normal saline as a lubricant). The time taken to secure the airway (from insertion to the first end-tidal carbon dioxide (ETCO_2_) tracing and five-point auscultation) and the number of attempts were noted. During extubation, blood staining was noted. In the postoperative period, sore throat was monitored with the numerical rating scale for the first 24 hours. Postoperative hoarseness of voice, cough and difficulty in swallowing were the other parameters noted. The results were entered in a standard spread sheet. Statistical analysis was done using JASP version 0.18.3.0 using the independent samples t-test and the Chi-square test for quantitative variables.

Results: All three groups were comparable in terms of demography (p > 0.05). The time taken to insertion, number of attempts and securing of the airway were also comparable amongst the three groups (p > 0.05). Blood staining during LMA removal was comparable between the three groups (p > 0.05). In the postoperative period, sore throat was comparable between the three groups (p > 0.05). The incidence of hoarseness of voice in the postoperative period however was more significant in Group LJ when compared to the other two (p < 0.05). Postoperative swallowing discomfort was also significantly higher in Group LJ than in the other two groups (p < 0.05).

Conclusion: We conclude that lignocaine jelly, water-based jelly and normal saline used as lubricating agents on the i-gel airway did not show a difference in the incidence of postoperative sore throat. Lignocaine jelly was associated with a higher incidence of hoarseness of voice and swallowing discomfort when compared to the other lubricants.

## Introduction

Laryngeal mask airways are an important part of the airway management protocol, widely used in both emergency situations and the operating room [1]. Being a device made of synthetic material, most supraglottic airway devices require some form of lubrication for their adequate placement without causing injury to the oropharynx [2]. This is crucial, especially in the operating room, where the effects of general anaesthesia and gas flow through circuits affect the function of salivary glands [3]. The i-gel® is a second-generation laryngeal mask airway that is known for its design to aid in quicker insertion and ensure a better seal over the larynx [4]. The recommended lubricant for the i-gel is the K-Y® Jelly, which is a water-based lubricant. However, due to easy availability in the setting of an operating room, lignocaine jelly is used in its place in many institutions. Hence, we designed this study to compare the effect of different lubricating agents on the incidence of sore throat, a common postoperative event with laryngeal mask airways [5].

## Materials and methods

This double-blind trial was registered with the Clinical Trial Registry of India (CTRI/2023/02/049480) and conducted over a six-month period at SRM Medical College and Hospital, from July 2023 to December 2023. After obtaining consent, 168 patients undergoing minor elective surgery requiring general anaesthesia at SRM Medical College and Hospital were initially enrolled in the study. The participants were aged between 18 and 70, belonged to both sexes, belonged to the American Society of Anesthesiologists Physical Status I and II and had Mallampati score grades 1 and 2. Patients with a history of sore throat within two weeks of the procedure, with known chronic obstructive airway disease or reactive airway diseases, with a diagnosis of gastro-oesophagal reflux disease or hiatus hernia, with an anticipated difficult airway with a restricted mouth opening (less than two fingers), a short neck and a body mass index of >30 kilograms per squared meter were excluded from the study.

Upon enrolment, the participants were allocated into three groups with 50 patients in each, through computer-generated random numbers based on the lubricant to be used: Group LJ with lignocaine jelly (LOX 2%: NEON ™), Group NS with normal saline (Fresenius Kabi™) and Group WJ with water-based jelly (Lubic: NEON™). Fasting guidelines and premedication were standardized for the three groups. On the day of the procedure, on arriving at the operating room, standard monitors were placed and preoxygenation was done. The opioid of choice was injection of fentanyl given at a dose of 2 micrograms per kilogram of body weight, while the induction agent of choice was injection propofol given at a dose of 2 milligrams per kilogram of body weight, while the induction agent of choice was injection propofol, given at a dose of 2 mg per kilogram of body weight, titrated to patient requirements. On attaining sufficient intubating conditions, an expert anaesthesiologist, with an experience of more than three years or who has had experience with more than 50 LMA insertions, placed the i-gel LMA coated immediately before insertion with the appropriate lubricating agent into the oropharynx of the patient based on the group to which they belonged. The parameter measured was the time to secure the airway that began with holding the i-gel LMA to its successful placement, confirmed by the first end-tidal carbon dioxide tracing on the monitor and five-point auscultation. A gaseous mixture of 2% weight by volume sevoflurane with 1 liter oxygen and 1 liter nitrous oxide was used for maintenance, and the patients were allowed spontaneous ventilation.

Patients requiring more than three attempts at insertion were considered failures, and standard general anaesthesia with endotracheal intubation was planned in the protocol in such a scenario. Patients undergoing procedures that lasted more than one hour were excluded from the study. At the end of the procedure, on removing the LMA, blood staining on the device surface was observed and made note of. The patients were then observed for a 24-hour period in the post-anesthetic care unit. A periodic re-evaluation was done every fourth hour, monitoring for sore throat measured through the numerical rating score (NRS), a scale of zero through 10 offered to the patient to mark. If the NRS was marked at more than six, injection fentanyl at one microgram per kilogram of body weight was administered. Any event of hoarseness of voice was measured on a scale of zero through three (0 - no hoarseness, 1 - minimal change in the quality of voice/affirmation on enquiry, 2 - moderate change in the quality of voice/patient complained on his own, 3 - gross change in quality of speech), and any complaints of cough were rated on a scale of zero through three (0 - no cough since surgery, 1 - minimal cough, 2 - moderate cough, 3 - severe cough [6]. Patients were also enquired and monitored for any event of swallowing discomfort and retching in the postoperative period.

Any other adverse events such as laryngospasm and bronchospasm were also noted. All the data were collected and compiled in a standard Excel sheet (MS Excel 2019, Microsoft Corporation, United States). Statistical analysis was done using the Chi-square and one-way ANOVA test using the IBM SPSS Statistics for Windows, Version 29.0 (released 2023, IBM Corp., Armonk, NY). Significance was taken when the p-value was <0.05

**Figure 1:**
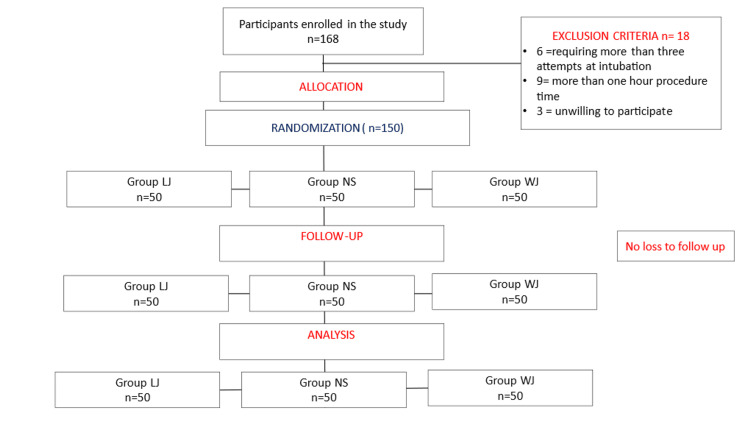
Consolidated Standards of Reporting Trials n = population in each group, NS = normal saline , LJ = lignocaine jelly, WJ = water-based jelly

The consolidated standards of reporting trials are mentioned in Figure 1.

## Results

In the present study, the patient characteristics and duration of surgery were comparable between the three groups (Tables 1, 2). In 127 patients, the i-gel was successfully placed at the first attempt (41 in Group LJ, 43 in Group NS and 43 in Group WJ), while 22 (eight in Group LJ, seven in Group NS and six in Group WJ ) required a second attempt for the successful placement of the i-gel. Two patients (one from the lignocaine jelly group and one from the water-based jelly group) required three attempts at intubation. This was not statistically significant. There was no incidence of insertion failure in all three groups (Table 1).

**Table 1:** Comparing qualitative patient characteristics amongst the three groups. LMA: laryngeal mask airway, ASA PS: American Society of Anesthesiologists Physical Status, n%: number percentage of the total population, MPC: Mallampati classification of airway. Group NS = group receiving normal saline as the lubricant, Group WJ = group receiving water-based jelly as lubricant, Group LJ = group receiving lignocaine jelly as the lubricant. Statistical analysis was done using the chi-squared test and p < 0.05 was considered significant. * - insignificant p-value. + - significant p-value

Demography		Group						p-value
		Group LJ		Group NS		Group WJ		
		Count	Column N %	Count	Column N %	Count	Column N %	
Sex	Male	27	54.0%	22	44.0%	23	46.0%	0.164*
	Female	23	46.0%	28	56.0%	27	54.0%	
MPC	Class I	21	42.0%	20	40.0%	22	44.0%	0.921*
	Class II	29	58.0%	30	60.0%	28	56.0%	
ASA PS	Class I	24	48.0%	25	50.0%	25	50.0%	0.974*
	Class 2	26	52.0%	25	50.0%	25	50.0%	
Size of LMA	Size 3	10	20.0%	9	18.0%	11	22.0%	0.882*
	Size 4	40	80.0%	41	82.0%	39	78.0%	
Attempts at insertion	1	41	82.0%	43	86.0%	43	86.0%	0.853*
	2	8	16.0%	7	14.0%	6	12.0%	
	3	1	2.0%	0	0.0%	1	2.0%	

**Table 2:** Comparing the quantitative patient characteristics and time taken to insert and secure the airway amongst the three groups. BMI: body mass Index, CM: centimeter, KG: kilogram, Min: minutes, Sec: seconds Group NS = group receiving normal saline as the lubricant, Group WJ = group receiving water-based jelly as a lubricant, Group LJ = group receiving lignocaine jelly as the lubricant, F = critical value. Statistical analysis was done using the one-way ANOVA test and p < 0.05 was considered significant. * - insignificant p-value. + - significant p-value

	Group						F	p-value
	Group LJ		Group NS		Group WJ			
	Mean	Standard Deviation	Mean	Standard Deviation	Mean	Standard Deviation		
Age (years)	42.54	12.64	44.06	12.42	43.20	10.50	0.174	0.840*
Height (cm)	167.12	5.84	167.04	5.92	166.04	6.61	0.481	0.618*
Weight (Kg)	65.82	6.52	66.62	6.11	64.60	6.53	1.267	0.284*
BMI	23.50	1.06	23.83	0.98	23.38	1.16	2.338	0.100*
Duration of procedure (min)	27.56	8.89	29.50	8.65	28.00	7.49	0.739	0.479*
Time taken for securing airway (sec)	16.94	6.16	15.80	3.87	16.38	5.61	0.577	0.562*

The time taken for securing the airway was not significantly different in the three groups: 16.94 ± 6.16 seconds vs. 15.80 ± 3.87 seconds vs. 16.38 ± 5.61 seconds in Group LJ, Group NS and Group WJ, respectively (p-value = 0.562) (Table 2).

Evaluation of sore throat in the postoperative period was done using the Numerical Rating Score. The patients were evaluated four hours once, till the first 24 hours. The incidence and severity of sore throat were similar between the groups and were statistically insignificant (p > 0.05). This shows that the three lubricants did not differ in terms of the incidence of sore throat (Table 3).

**Table 3:** Comparing the Numerical Rating Scores of patients between the three groups over a 24-hour period, at four-hour intervals. NRS: Numerical Rating Score Group NS = group receiving normal saline as the lubricant, Group WJ = group receiving water-based jelly as the lubricant, Group LJ = group receiving lignocaine jelly as the lubricant. Statistical analysis was done using the chi-square test and p < 0.05 was considered significant * - insignificant p-value. + - significant p-value NRS 0 signifies the baseline numerical rating score on the immediate removal of the i-gel device. NRS followed by the subsequent numbers suggests the hour at which the next score analysis was taken.

Numerical Rating score at every four hour Interval for the first 24 hours	Average score	GROUPS						p-value
		GROUP LJ		GROUP NS		GROUP WJ		
		Count	Column N %	Count	Column N %	Count	Column N %	
NRS 0	0	40	80.0%	41	82.0%	42	84.0%	0.681*
	2	8	16.0%	9	18.0%	7	14.0%	
	4	2	4.0%	0	0.0%	1	2.0%	
NRS 4	0	45	90.0%	45	90.0%	44	88.0%	0.664*
	2	4	8.0%	5	10.0%	6	12.0%	
	4	1	2.0%	0	0.0%	0	0.0%	
NRS 8	0	46	92.0%	50	100.0%	49	98.0%	0.224*
	2	3	6.0%	0	0.0%	1	2.0%	
	4	1	2.0%	0	0.0%	0	0.0%	
NRS 12	0	48	96.0%	50	100.0%	50	100.0%	0.132*
	2	2	4.0%	0	0.0%	0	0.0%	
NRS 16	0	48	96.0%	50	100.0%	50	100.0%	0.132*
	2	2	4.0%	0	0.0%	0	0.0%	
NRS 20	0	49	98.0%	50	100.0%	50	100.0%	0.365*
	2	1	2.0%	0	0.0%	0	0.0%	
NRS 24	0	49	98.0%	50	100.0%	50	100.0%	0.365*
	2	1	2.0%	0	0.0%	0	0.0%	

Other parameters monitored for were hoarseness of voice, swallowing discomfort and cough. The number of patients with no complaints of hoarseness of voice was 47 (94%) in Group NS, 49 (98%) in Group WJ and 32 (64%) patients in Group LJ. This was found to be significant (p < 0.05). In terms of severity, three patients from Group NS (6%), one patient from Group WJ (2%) and fourteen patients (28%) in Group LJ group had complaints of mild hoarseness of voice. Around four patients (8%) in Group LJ had moderate hoarseness of voice, while there were no such complaints in Groups NS and WJ. This difference in the severity of hoarseness was found to be statistically significant with a p-value < 0.05 (Table 4).

**Table 4:** Comparing the postoperative complications between the three groups. The values were recorded in the immediate postoperative period on removing the i-gel device. PONV: postoperative nausea and vomiting. Comparing the postoperative complications between the three groups: Group NS = group receiving normal saline as the lubricant, Group WJ = group receiving water-based jelly as the lubricant, Group LJ = group receiving lignocaine jelly as the lubricant. Statistical analysis was done using the chi-square test and p < 0.05 was considered significant * - insignificant p-value. + - significant p-value. Grades of hoarseness: three (0 – no hoarseness, 1 – minimal change in the quality of voice, 2 – moderate change in the quality of voice, 3 – gross change in the quality of speech). Grades of cough: (0 – no cough since surgery, 1 – minimal cough, 2 – moderate cough, 3 – severe cough)

Postoperative complications		Group						P-value
		Group LJ		Group NS		Group WJ		
		Count	Column N %	Count	Column N %	Count	Column N %	
Sore throat	Yes	9	18.0%	10	20.0%	7	14.0%	0.722*
	No	41	82.0%	40	80.0%	43	86.0%	
Cough	Grade 0	44	88.0%	39	78.0%	41	82.0%	0.413*
	Grade 1	6	12.0%	11	22.0%	9	18.0%	
Hoarseness	Grade 0	32	64.0%	47	94.0%	49	98.0%	<0.0001+
	Grade 1	14	28.0%	3	6.0%	1	2.0%	
	Grade 2	4	8.0%	0	0.0%	0	0.0%	
Blood staining	No	49	98.0%	50	100.0%	49	98.0%	0.602*
	Yes	1	2.0%	0	0.0%	1	2.0%	
Swallowing discomfort	No	28	56.0%	46	92.0%	48	96.0%	<0.0001+
	Yes	22	44.0%	4	8.0%	2	4.0%	
PONV	No	40	80.0%	46	92.0%	50	100.0%	0.003+
	Yes	10	20.0%	4	8.0%	0	0.0%	

On assessing swallowing in these patients, 22 (44%) patients in Group LJ complained of discomfort, while only four (8%) patients in Group NS and two (4%) patients in Group WJ had such complaints. This was significant with a p < 0.001 (Table 4).

On comparing any event of blood staining on the surface of the i-gel LMA at the end of procedure, one patient in Group LJ and one patient in Group WJ had trace staining during the LMA removal. This did not carry any statistical significance, i.e., p > 0.05 (Table 4).

Around six patients in Group LJ, 11 patients in Group NS and nine patients in Group WJ had complaints of cough in the postoperative period. This was insignificant with a p-value > 0.05 (Table 4).

## Discussion

As the predecessor to all supraglottic airway devices, the laryngeal mask airway (LMA) is an important tool in the armamentarium of airway equipment, serving as a bridge between the endotracheal tubes and face masks [7]. While they offer several advantages over traditional endotracheal tubes, the morbidity associated with such LMA devices is seen in the form of minor complications such as sore throat, soft tissue injury during insertion, hoarseness and dysphagia. Lubricants are essential prior to placement of LMAs. This is because routine anticholinergic use and the high flow rates of anaesthetic gases tend to decrease salivary gland function [8,9]. Numerous LMAs have been developed over the years to enable ease of insertion while tackling the many disadvantages. The i-gel LMA® developed by Intersurgical™ is a second-generation true anatomic, pre-shaped, single-use device that exactly mirrors the laryngeal architecture. The i-gel is made of a medical-grade thermoplastic elastomer. The tacky nature of the material requires the i-gel LMA to be adequately lubricated before placing into the oral cavity. The recommended lubricant is the K-Y® water-based jelly. However, due to its limited availability, 2% lignocaine jelly is commonly used, given that it is invariably present in all operating rooms (Table 5).

**Table 5:** Comparing the different agents of lubrication commonly used and available in terms of the chemical composition and price with the human saliva.

	Water-based jelly	Normal saline	2% lignocaine jelly	Saliva
Lubricant manufacturer	Lubic (NEON)™	NS (Fresenius Kabi)™	LOX 2% (NEON)™	98.5 % water mucin enzymes, 1% organic substances, 0.5% inorganic substances
Composition	Glycerin propylene glycol gluconolactone, methylparaben propylparaben water-soluble gel base in purified water	Each 100 ml contains 154 mmol/litre sodium 154 mmol/litre chloride 308 mmol/l osmolality.	Lignocaine hydrochloride 2% w/v, Each ml 20 mg lignocaine, methylparaben 0.061% w/v, propyl paraben 0.027% w/v, water-soluble gel base	
pH	pH 4.5	pH 5.5	pH 6-7	pH 6.02- 7.05
Price	INR 5.6/g	INR 39.04	INR 1.25/g	

Lignocaine jelly is a commonly used topical local anaesthetic. Keeping its anaesthetic properties in mind, this study was conducted to see if this would benefit in the postoperative period with decreased incidences of sore throat, hoarseness of voice, swallowing discomfort and cough [8,9]. However, we observed that the incidence of postoperative sore throat was similar between the three groups of lubricating agents. The results of our study were comparable to those of the study by Park et al., where normal saline was compared to lignocaine jelly and the incidence of sore throat did not vary [10]. Similarly, a study by Keller et al. also showed that the lubricants did not have an advantage over each other in producing sore throats [11].

We found that the incidence of hoarseness of voice and swallowing discomfort was significantly higher in the patients where lignocaine jelly was used as a lubricating agent than in patients in whom water-based jelly or normal saline was used. While aerosolized lignocaine is found to have more episodes of hoarseness of voice, the effect of lignocaine jelly has not been assessed clearly to date [12]. This study shows that patients receiving lignocaine jelly as a lubricant had mild hoarseness of voice in the immediate postoperative period, which did not require any intervention. The results of our study are similar to those of Sumathi et al., who suggest that better agents of lubrication, such as betamethasone jelly, might prove to have better benefits [13]. This suggests that lignocaine jelly, due to its bitter nature, density and anaesthetic properties, causes a numbing effect on the laryngopharynx that could account for the hoarseness of voice and swallowing discomfort [14,15]. 

Complaints of cough in the immediate post-surgery period were comparable amongst the three groups. This implies that even though there is a theoretical probability of the loss of the cough reflex on applying lignocaine jelly to the oropharynx, the effect was not potent enough to completely obscure the cough reflex [16,17].

The incidence of blood staining on the surface of the supraglottic airway device was similar between the three groups, which suggests that other factors like the attempts at insertion or an unanticipated difficult airway could contribute to this particular side effect [18,19].

Our study shows that lignocaine jelly does not offer any advantage as a lubricating agent over the recommended water-based jelly. Our study shows similar results to a study by Doukumo et al., who studied the effect of different lubricating agents on endotracheal intubation [20].

One patient in Group WJ and two patients in Group NS had an episode of vomiting. Injection ondansetron 4 milligrams was given after re-assuring the patients.

Limitations

Although the degree of hoarseness of voice and presence of swallowing discomfort were evaluated in the immediate postoperative period by us, we did not follow through the entire 24 hours to check its resolution. Furthermore, the postoperative changes in the pharyngeal mucosa through endoscopic view could not be assessed by us. This can be a prospect in future studies.

## Conclusions

We conclude that the three lubricating agents, namely, lignocaine jelly, water-based jelly and normal saline, did not have an advantage over one another in reducing the incidence of postoperative sorethroat. On screening these patients for other side effects , we noticed higher rates of hoarseness of voice and swallowing discomfort with patients who received lignocaine jelly as the lubricant of choice . Further studies with a larger sample size can aim at understanding the effect that different agents have on the oral mucosa , especially at finding the most suitable agent for lubrication.
